# Case Report: Mitral Valve Involvement and First-Degree Atrial-Ventricular Block in Two Patients With Multisystem Inflammatory Syndrome in Children

**DOI:** 10.3389/fped.2021.676934

**Published:** 2021-08-06

**Authors:** Paola Di Filippo, Massimiliano Raso, Marta Cacciatore, Roberta Patacchiola, Giulia Renda, Nadia Rossi, Francesco Chiarelli

**Affiliations:** ^1^Department of Pediatrics, University of Chieti, Chieti, Italy; ^2^Department of Cardiology, University of Chieti, Chieti, Italy

**Keywords:** COVID-19, MIS-C multisystem inflammatory syndrome in children, child, SARS–CoV−2, heart involvement

## Abstract

COVID-19 seems to be less frequent and severe in children compared to adults. Despite the very few symptoms usually found in children, great attention was recorded when in April 2020 a hyperinflammatory process in children with fever and multiorgan involvement after a paucisymptomatic COVID infection was reported. The United States Centers for Disease Control and the World Health Organization recognized and defined this syndrome as “Multisystem Inflammatory Syndrome in Children (MIS-C).” We describe two cases of MIS-C presenting with fever, cutaneous rash, and a mild cardiac involvement expressed with a transient mitral valve involvement and a first-degree atrioventricular block. Acute treatment was managed with intravenous immunoglobulin, oral aspirin, and intravenous corticosteroids reaching consequent good outcome. Clinical characteristics, treatment management, follow-up, and long-term evolution of children with MIS-C are still poorly defined. Further research is needed to better understand the pathogenesis of this newly described condition, to validate a high-level recommended therapy and a specific therapy tapering timings.

## Background

COVID-19 resulted to be less frequent and severe in children compared to adults, involving 1–5% of the COVID-19 cases (1) and requiring intensive care unit admission in 0.6–2% of cases (2). The low infection rate in the pediatric population is probably related to the generally paucisymptomatic course of the infection, which consequently receives less medical attention (3, 4).

Despite the very few symptoms usually found in children, greater attention was recorded when in April 2020 an English child showed an abnormal systemic inflammatory response with fever and multiorgan involvement after a paucisymptomatic SARS-CoV-2 infection (5). This condition was first called “Pediatric Inflammatory Multisystem Syndrome Temporally associated with Severe acute respiratory syndrome coronavirus 2” (PIMS-TS) (6). Similar cases in Europe and in the US were described, and they were associated temporally and geographically with COVID-19 outbreaks (5, 7, 8).

Therefore, the United States Centers for Disease Control (CDC) (9) and the World Health Organization (WHO) (10) recognized and defined this syndrome by renaming it “Multisystem Inflammatory Syndrome in Children (MIS-C) associated with COVID-19.” The two definitions proposed by the CDC and the WHO are both based on the clinical presentation (fever, skin involvement, gastrointestinal symptoms), the multiorgan involvement, the absence of a plausible alternative diagnosis, current or recent SARS-CoV-2 infection, or an exposure to positive patients within 4 weeks from the onset of symptoms. The definition proposed by the WHO also includes laboratory inflammatory markers.

To date, more than 1,000 cases were reported worldwide in the pediatric population (11, 12), but the real incidence of MIS-C is not yet known. Below we report two cases of children suffering from MIS-C with transient mitral valve involvement and arrhythmias.

### Case 1

A previously healthy 6-year-old male was admitted to the Pediatric Department for a 2-day fever (maximum body temperature: 38.6°C), fatigue, and loss of appetite. Six weeks earlier, he suffered from an asymptomatic SARS-CoV-2 infection documented by the nasopharyngeal swab. After 15 days from the onset, the nasopharyngeal swab negativity and IgG antibodies appearance were documented.

**Figure 1:**
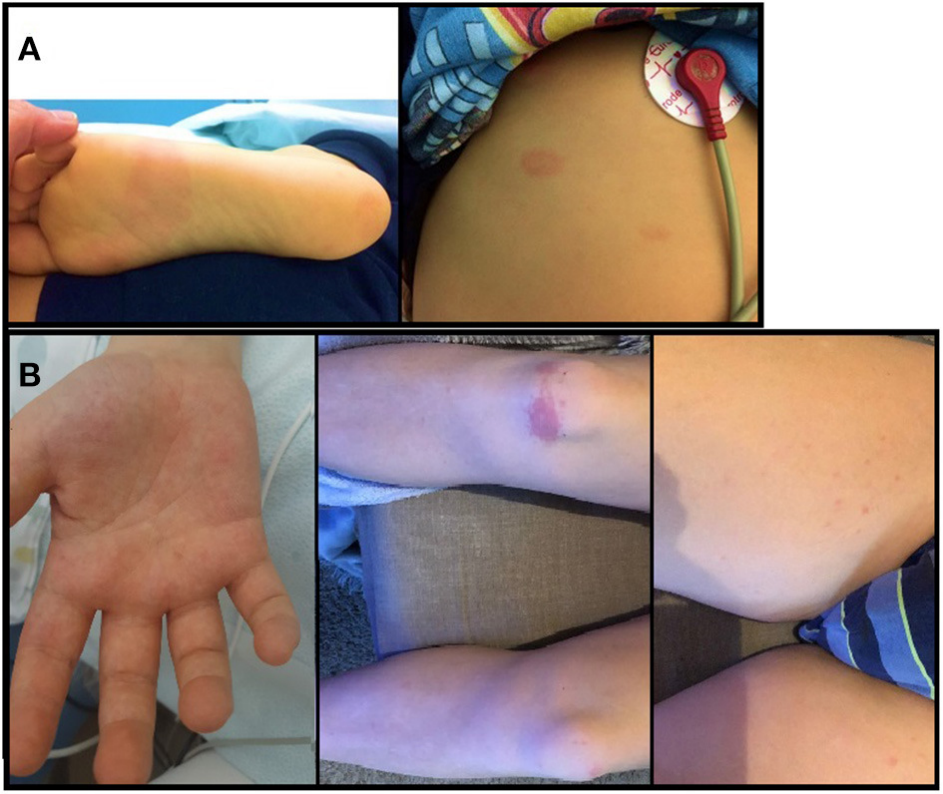
**(A)** non-itchy macular erythematous rash on the soles of the feet and trunk of the first patient; **(B)** non-itchy erythematosus rash on palms of the hands, on soles of the feet, on trunk and limbs of the second patient.

**Figure 2:**
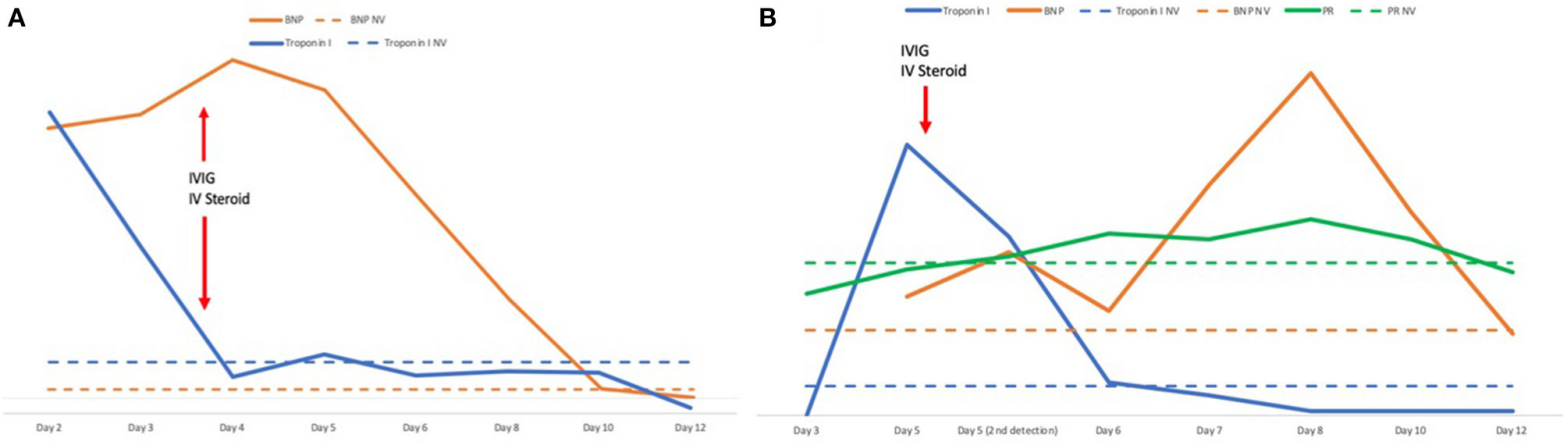
**(A)** Timeline of tropinin and BNP levels and therapy with IVIG and steroids in case 1. **(B)** Timeline of troponin, BNP levels, PR interval and therapy with IVIG and steroids in case 2. The number of days refers to the onset of the fever. BNP, brain natriuretic peptide; IVIG, intravenous immunoglobulins; IV, intravenous; NV, normal values.

The child appeared dehydrated and asthenic, with a non-itchy macular erythematous rash on the soles of the feet, ankles, and trunk (Figure 1A); furthermore, he had a body temperature equal to 40°C with tachycardia (heart rate 150 beats per minute) and hypotension (blood pressure 96/45 mmHg) but no desaturation (oxygen saturation 99%). Blood tests showed normal white blood cell count (7,760/μl) with lymphopenia (L: 710/μl) and increased inflammatory markers (C-reactive protein CRP: 89.7 mg/l, normal range: 0–5; procalcitonin PCT: 8.9 ng/ml, normal range: 0.02–0.5). In addition, a hyponatremia (126 mmol/l, normal range: 135–145) and an increase in cardiac markers (troponin I 201.5 pg/ml, normal range: 0–34.2; brain natriuretic peptide BNP 2,782 pg/ml, normal range 10–100; Figure 2A), amylase and lipase (145 U/l and 166 U/l, respectively, normal range 8–65), D-dimer (1.5 mg/l, normal range 0–5), IL-6 (201.6 pg/ml, normal range < 6.4), and ferritin (301 ng/ml, normal range 22–274) were found. Chest X-ray was normal. A 12-lead electrocardiography (ECG) was normal, and an echocardiogram found normal ventricular function with ejection fraction equal to 60%, and minimal mitral and tricuspid regurgitation. Rehydrating intravenous therapy and antibiotic therapy with ceftriaxone were started.

Twenty-four hours later, the patient was still febrile; tachycardia, hypotension, and cutaneous rash on the trunk persisted and a mild eyelid swelling appeared. The blood test showed persistence of hyponatremia (131 mmol/l), an increase in BNP (3,486 pg/ml), CRP (109.5 mg/l), and a reduction of troponin I (124.6 pg/mL; Figure 2A). An ECG control found a T-wave inversion in the anterior precordial leads. Blood, stool, and urine cultures were negative.

**Figure 3:**
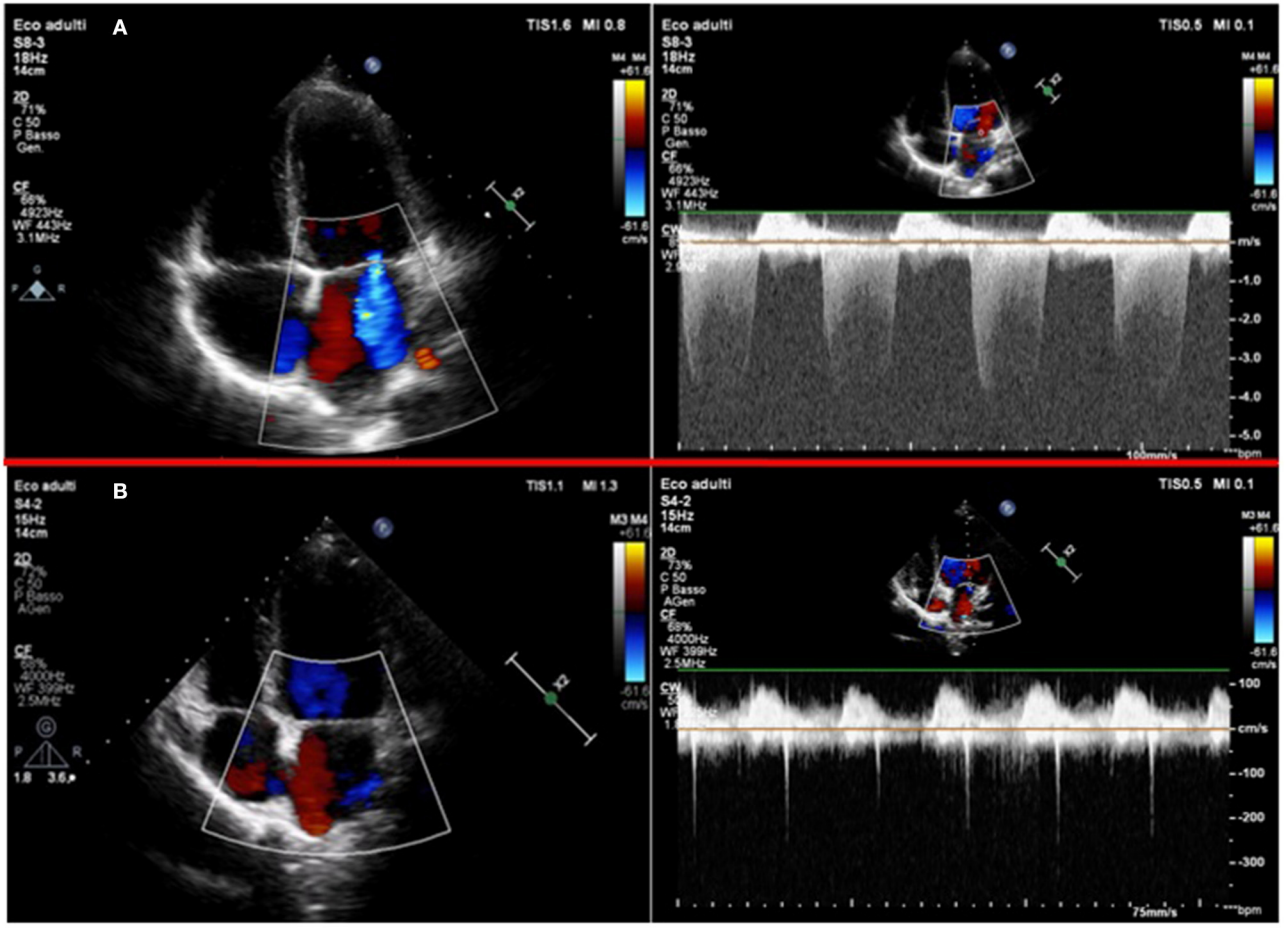
**(A)** 2-D Color-Doppler echocardiography of the first patient in the acute phase: left panel shows the apical 4 chambers view with color flow Doppler, showing a moderate mitral regurgitation; right panel shows the continuous wave Doppler of the mitral regurgitant jet. **(B)** 2-D Color-Doppler echocardiography of the same patient 1 month after: left panel shows an apical 4 chambers view with color flow Doppler and right panel shows the continuous wave Doppler of the minimal mitral regurgitation.

**Figure 4:**
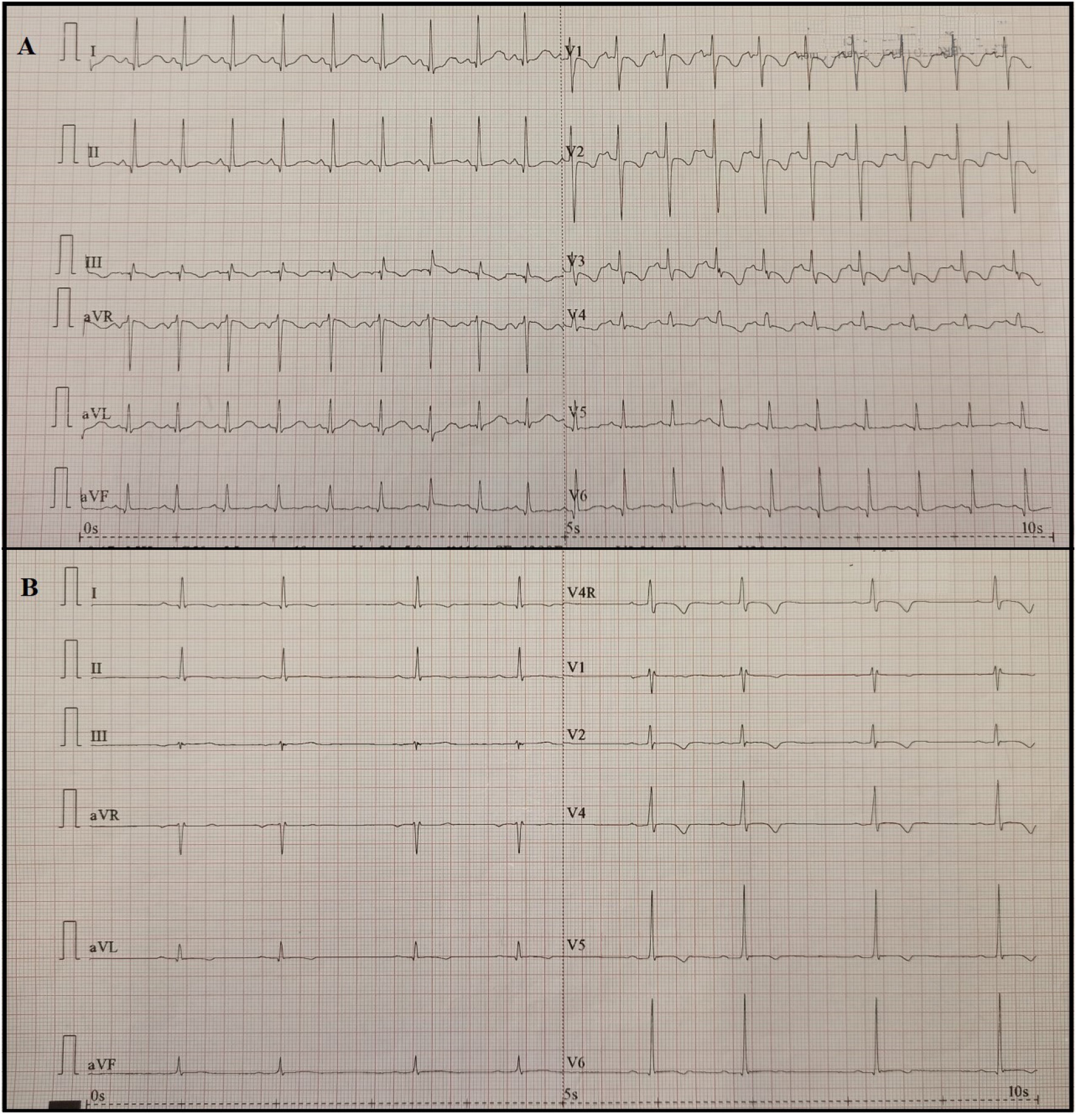
**(A)** 12-lead ECG of the first patient 5 days after fever onset showed a mild ST-segment depression and a deeper T-wave inversion compared with previous ECG in the anterior precordial leads; **(B)** 12-lead ECG of the second patient 8 days after fever onset showed a sinus bradycardia (HR 48 beats per minute) with a first-degree atrioventricular block (PR = 216ms).

A diagnosis of MIS-C was established, and intravenous immunoglobulins (IVIG) at the dosage of 2 g/kg in 48 h and methylprednisolone at the dosage of 2.25 mg/kg/day were started. About 24 h after, the child's clinical condition improved considerably. Fever and cutaneous rash disappeared, and blood pressure and heart rate improved (100/65 mmHg and 81 beats per minute, respectively). The blood test showed a normalization of sodium and troponin I levels (138 mmol/l and 28.2 pg/ml, respectively), and a reduction of BNP, CRP, and PCT levels (1,025 pg/ml, 18.36 mg/l, 0.68 ng/ml, respectively). However, 5 days after the fever onset the echocardiogram showed a “mild tricuspid regurgitation and moderate mitral regurgitation” (Figure 3A) and the ECG found a QT prolongation, equal to 492 ms (Figure 4A).

Following laboratory controls documented inflammation indices and cardiac enzymes in the normal range; however, increased platelet count was found (PLT: 1,250,000/mmc) and therapy with acetylsalicylic acid (ASA) at the dosage of 81 mg/day was then administered. Methylprednisolone was continued for 10 days with gradual tapering and then replaced with oral prednisone for 10 days with gradual tapering.

The patient was discharged with a clinical, cardiac, and radiological follow-up program. ASA therapy was continued until normal coronary artery confirmation at ≥4 weeks after diagnosis; complete normalization of the platelet count was documented 23 days after the onset of the syndrome.

At follow-up, ECG was normal and the echocardiogram 30 days after the onset of the syndrome showed normal ventricular function and the resolution of mitral regurgitation (Figure 3B). About 40 days after the onset, a cardiac magnetic resonance imaging (MRI) was performed before and during gadolinium administration revealing no morpho-functional abnormalities with non-dilated ventricles; a mild pericardial effusion with a maximum thickness of 7 mm (Supplementary Figure 1); normal systolic function; and no transvalvular flow changes with cine b-SSFP sequences.

### Case 2

A previously healthy 12-year-old boy was admitted to the Pediatric Department with a 2-day history of fever, myalgia, and non-itchy erythematosus rash on palms of the hands, on soles of the feet, on trunk, and on limbs (Figure 1B). Five weeks earlier, the patient had pauci-symptomatic COVID-19, confirmed with the SARS-CoV-2 RT-PCR test. After 26 days from the onset, the nasopharyngeal swab negativity and IgG antibody appearance were documented.

Blood tests documented lymphopenia (white blood cells 6,180/μl, lymphocytes 660/μl); an increase in inflammatory indices (CRP 70.4 mg/l, PCT 1 ng/ml), D-dimer (1.25 mg/l), and fibrinogen (550 mg/dl); and a mild hyponatremia (134 mmol/l). The arterial blood gas test was normal. The 12-lead ECG found a T-wave inversion in the anterior precordial leads with negative troponin I. Chest X-ray was normal.

Rehydrating intravenous therapy and antibiotic therapy with ceftriaxone were started. After 4 days of therapy-resistant fever, abdominal pain and vomiting also appeared. Control blood tests documented further increased inflammatory indices (CRP 166 mg/l, PCT 4.1 ng/ml), fibrinogen (640 mg/dl), ferritin (521 ng/ml), IL-6 (70.7 pg/ml), and a worsening hyponatremia (132 mmol/l). An increase in troponin I (321 pg/ml) and BNP (273 pg/ml) was documented (Figure 2B). Increases in ferritin (521 ng/ml), IL-6 (70.7 pg/ml), triglyceride (171 mg/dl, normal range 0–150), and liver enzyme (AST 156 U/l, normal range 5–34; ALT 175 U/l, normal range 0–55) values were also shown. The echocardiography documented “ejection fraction equal to 63%, minimal mitral and tricuspid regurgitation, and pericardial layers detachment.” The abdomen ultrasound was negative. Blood, stool, and urine cultures were negative so antibiotic therapy with ceftriaxone was interrupted. No acute infections with Epstein–Barr virus, cytomegalovirus, parvovirus B19, mycoplasma, coxsackievirus, echovirus, influenza A and B, adenovirus, and parainfluenza virus were found.

Since clinical and laboratory characteristics were compatible with MIS-C, therapy with IVIG (1.8 g/kg) in two consecutive days, ASA (81 mg/die), intravenous methylprednisolone (1.8 mg/kg/day), and oral proton-pump inhibitor with lansoprazole were started.

Defervescence and improved clinical condition were documented, but 8 days after fever onset the echocardiography found worsening ejection fraction (53%) and moderate mitral regurgitation in association with increased BNP (405 pg/ml, Figure 2B) and oliguria. In addition, flat and inverted diffusely T waves and bradycardia (heart rate 40 beats per minute at night, 55 beats per minute during the day) with PR interval prolongation (from 188 ms at the onset, PR reached a maximum of 232 ms 8 days after the onset) and a first-degree atrioventricular block (AVB) appeared (Figure 4B). Holter monitor confirmed bradycardia and first-degree AVB, with a worsening in the nocturnal period probably due to vagal hypertonus.

During the hospitalization, troponin I and BNP values showed progressive decrease (Figure 2B). The normalization of troponin was documented 6 days after the onset of symptoms. Twelve days after the fever onset, BNP in the normal range and a normal ECG with the AVB disappearance were found; furthermore, the echocardiogram documented preserved global systolic function (ejection fraction 65%), slight hyperreflective papillary muscles, and minimal mitral regurgitation in the absence of pericardial effusion. Given the clinical, laboratory, and instrumental improvement, methylprednisolone therapy was tapered. About 20 days after the fever onset, a cardiac MRI was performed before and during gadolinium administration and it showed no morpho-functional abnormalities with non-dilated ventricles; normal systolic function; no myocardial edema or intracavitary thrombotic formations; and no transvalvular flow changes with cine b-SSFP sequences.

The patient was discharged with a clinical and cardiac follow-up program. ASA therapy was continued until normal coronary artery confirmation at ≥4 weeks after diagnosis, and steroid therapy was gradually tapered along 1 month.

## Discussion

MIS-C is a recent recognized spectrum of disease manifestations in children associated with SARS-CoV-2 infection (13). However, few cases were reported in literature so far.

The clinical characteristics of children with MIS-C are still poorly defined (14). The most frequent symptoms at the onset of the disease are fever, gastrointestinal symptoms (vomiting, abdominal pain, diarrhea), cutaneous rash, respiratory symptoms (cough and dyspnea), and mucosal changes (15). Cardiovascular symptoms were described in about 70% of cases (14), and heart involvement is the most feared complication. A ventricular systolic dysfunction was found in about 55% of patients at the time of discharge with a mortality of < 2% (16).

MIS-C clinical manifestations often overlapped with Kawasaki disease (fever, rash, conjunctival injection) (16, 17), but patients with MIS-C are typically older, involving more frequent children between 5 and 9 years of age (5, 15, 18). Our patients were 6 and 12 years old, and their main clinical manifestations were fever, cutaneous rash, and a mild cardiac involvement. In fact, although early reports of MIS-C all involved cardiovascular shock, cardiovascular collapse is not a requirement for diagnosis (19). Neither respiratory symptoms nor mucosal changes were reported in our patients, but a mild increase in pancreatic enzymes in the first child and vomiting in the second child were documented.

The most frequent laboratory findings are an increase in inflammatory indices (CRP, procalcitonin, ferritin, ERS, IL-6), cardiac markers (BNP, troponin), and D-dimer levels, leukocytosis (with lymphopenia), and reduced albumin levels (12, 16). According to the CDC, positivity for SARS-CoV-2 antibodies was documented in 98% of 1,097 confirmed cases of MIS-C (12). These laboratory findings were all recognized in our patient, except for reduced albumin levels.

Patients with MIS-C require real-time assessment, cardiology reassessment, and imaging frequency depend on disease severity. Regular cardiac testing includes serial ECG, echocardiography, and rhythm monitoring, with consideration of cardiac MRI and exercise stress testing as clinically indicated. McMurray et al. (16) suggest more frequent echocardiography assessment during the 1st week of presentation, then spacing the frequency once the clinical trajectory is better established. The most frequent echocardiographic findings in MIS-C patients are left ventricular systolic dysfunction with decreased ejection fraction, coronary aneurysms, or dilatation and pericardial effusion (15). In the first case, a worsening mitral regurgitation and a QT prolongation 5 days after the fever onset were found. Although valve damage is included in the WHO diagnostic criteria (9), few cases of mitral regurgitation without ventricular dysfunction in MIS-C-affected children were reported. Blondiaux et al. (20) found a mitral valve regurgitation related to the left ventricle dilatation during the acute phase of the disease in two children. In our patient, the serial echocardiographic controls detected the worsening valve damage that did not reflect the clinical and laboratory improvement of the patient.

The pathogenesis of cardiac damage in MIS-C is not yet known. Recent studies hypothesized that high levels of interleukin-6 and interleukin-1β may play a key role in triggering a cytokine storm and consequent myocardial damage (15, 16). As in the pathogenesis of valvular damage in rheumatic carditis (21), we speculate that the cytokine storm might cause inflammation in the connective tissue of the mitral valve of our patient with a complete restitutio ad integrum of the valve function after complete resolution of the inflammatory process.

In the second case, a worsening ejection fraction and mitral regurgitation associated with a first-degree AVB appeared 7 days after fever onset. Myocarditis is the main cause of AVB in young and middle-aged patients (22), and conduction abnormalities were also observed during the SARS-CoV outbreak in 2002–2003 (23). Cardiac arrhythmias were reported in 16.7% out of 138 hospitalized COVID-19 patients in a retrospective single-center case series (24), and an asymptomatic AVB was found in subclinical myocarditis without extensive systemic or myocardial involvement (25).

It is not yet understood if arrhythmia and AVB in particular are due to direct viral involvement or to an exaggerated inflammatory response. However, many cases of arrhythmia during MIS-C several weeks after the infection acute phase suggest that the underlying mechanism could be the myocardium local inflammation (26) due to a high systemic inflammatory response *via* cytokine release (interleukin-6 and tumor necrosis factor-a) (27) causing edema of the conduction tissue (28).

In a recent series including 25 children with MIS-C, ECG anomalies were found in 56% of children and included PR and QTc prolongation and ST segment changes. First-degree AVB was found in 20% (5/25) of children at a median of 6 days after fever onset, and four out of five patients evolved to second- or third-degree AVB (29). Differently from non-COVID-19-related myocarditis, no patients with MIS-C-related AVB required acute resuscitation, pacing, or medication. First-degree AVB resolved between 10 and 14 days after the fever onset in four of five children, and higher-grade AVB resolved within about 1 week (29). Therefore, ECGs should be monitored for evidence of PR prolongation, especially in children who develop first-degree AVB because of the risk of progression to high-grade AVB.

A rapid diagnostic setting is important to promptly direct appropriate therapy to prevent long-term sequelae such as coronary dilatation, fibrosis, and scarring of the heart and to reduce the chance of being admitted to the intensive care unit (30).

To date, no comparative studies on the different therapeutic approaches to the disease were performed. The current therapeutic recommendations proposed by the American College of Rheumatology (30) and the Rheumatology Study Group of the Italian Society of Pediatrics (31) are based on therapeutic regimens for Kawasaki disease and recommendations from professional societies. The current treatment protocols can be grouped under four categories (supportive, antibiotics, anti-inflammatory, anticoagulant). The supportive care consists of respiratory support and maintenance of fluid electrolyte balance and blood pressure. Broad-spectrum antibiotics empirically treat potential bacterial infections. The anti-inflammatory management consists of high-dose intravenous immunoglobulin (IVIG) at 2 g/kg (30, 32). Anti-inflammatory treatment aims to reduce tissue inflammation to prevent or limit the progression of coronary aneurysm and cardiac injury reducing significantly cytokines, monocytes, macrophages, neutrophils, and activated T cells and increasing natural killer cells (33). Similarly to Kawasaki disease, in MIS-C patients IVIG administration is followed by a rapid resolution of symptoms (32) and a considerable clinical and laboratory improvement was also found in our patient after IVIG administration. Antiplatelet agents are also recommended, given the risk of thrombosis associated with coagulation abnormalities in patients with MIS-C (increased D-dimer fibrinogen and platelets) (30).

Corticosteroids and biologic response modified such as Anakinra (IL-1 receptor antagonist) should be considered under the guidance of rheumatology consultation. The goal is to control and eliminate inflammation as quickly as possible before the onset of tissue damage (34).

Lastly, anticoagulation with enoxaparin or warfarin is indicated in MIS-C patients with a coronary artery z-score >10.0 or moderate/severe left ventricular dysfunction (EF <35%) (30).

About our experience, ASA therapy was continued for 28 days in both patients, until it was proven that there were no coronary artery aneurysms 4 weeks after the disease onset, even if platelet count normalization occurred at 23 days in the first patient. After the initial methylprednisolone administration, a progressive tapering of oral prednisone for ~2–4 weeks was done.

Although ventricular dysfunction appears to resolve quickly in most MIS-C patients, the long-term complications of myocardial inflammation are unknown and may include myocardial fibrosis and scarring. For this reason, cardiac MRI should be performed 2–6 months after the acute event in all patients with abnormal ventricular function (30). Cardiac MRI of our patients did not find signs of fibrosis or scarring. So, early diagnosis and early adequate therapy may positively impact long-term outcomes.

In conclusion, knowledge about this pathology is still very poor. These cases illustrate that MIS-C can develop despite SARS-CoV-2 infection severity and highlight the importance of the awareness of this new entity for a promptly diagnosis and treatment to avoid cardiac sequelae as much as possible.

Although cardiac involvement is common, not all MIS-C cases are characterized by severe clinical features requiring intensive care and these patients can often be managed in pediatric wards. However, children with MIS-C should perform frequent echocardiogram to detect valve and myocardial damage and frequent ECGs to monitor for PR prolongation and atrioventricular conduction disease, even at follow-up after discharge.

Unfortunately, long-term outcomes of heart involvement were not yet known. Follow-up will permit to clarify the long-term evolution and to stratify patients. Further research is needed to better understand the pathogenesis of this newly described condition, to validate a high-level recommended therapy and a specific therapy tapering timings.

## Data Availability Statement

The raw data supporting the conclusions of this article will be made available by the authors, without undue reservation.

## Author Contributions

PD coordinated the case report, drafted, and created figures. MC and MR drafted the manuscript. NR drafted part of the manuscript and reviewed the manuscript. RP created figures. GR helped for the cardiology content. RP, NR, and FC reviewed critically the manuscript. All authors contributed to the article and approved the submitted version.

## Conflict of Interest

The authors declare that the research was conducted in the absence of any commercial or financial relationships that could be construed as a potential conflict of interest.

## Publisher's Note

All claims expressed in this article are solely those of the authors and do not necessarily represent those of their affiliated organizations, or those of the publisher, the editors and the reviewers. Any product that may be evaluated in this article, or claim that may be made by its manufacturer, is not guaranteed or endorsed by the publisher.

## Supplementary Material

Supplementary Figure 1 The cardiac magnetic resonance imaging performed about 40 days after the onset found no morpho-functional abnormalities with non-dilated ventricles, a normal systolic function and a mild pericardial effusion with a maximum thickness of 7 mm.

## Abbreviations

COVID-19: Corona Virus Disease 19
MIS-C: Multisystem Inflammatory Syndrome in Children
SARS-CoV-2: severe acute respiratory syndrome coronavirus 2
PIMS-TS: Pediatric Inflammatory Multisystem Syndrome Temporally associated with Severe acute respiratory syndrome coronavirus 2
CDC: Centers for Disease Control
WHO: World Health Organization
CRP: C-reactive protein
PCT: procalcitonin
BNP: brain natriuretic peptid
ECG: electrocardiography
IVIG: intravenous immunoglobulins
ASA: acetylsalicylic acid
MRI: magnetic resonance imaging
ERS: Erythrocyte sedimentation rate
IL: Interleukin
AVB: atrioventricular block.

## References

[1] Ludvigsson JF. Systematic review of COVID-19 in children shows milder cases and a better prognosis than adults. Acta Paediatr. (2020) 109:1088–95. 10.1111/apa.15270 32202343 PMC7228328

[2] Wu Z McGoogan JM. Characteristics of and important lessons from the coronavirus disease 2019 (COVID-19) outbreak in China: summary of a report of 72314 cases from the Chinese Center for Disease Control and Prevention. JAMA. (2020) 323:1239–42. 10.1001/jama.2020.2648 32091533

[3] Hatmi ZN. A systematic review of systematic reviews on the COVID-19 pandemic. Clin Med. (2021) 26:1–18. 10.1007/s42399-021-00749-y 33521564 PMC7835449

[4] Mehta NS Mytton OT Mullins EWS Fowler TA Falconer CL Murphy OB. SARS-CoV-2 (COVID-19): what do we know about children? A systematic review. Clin Infect Dis. (2020) 71:2469–79. 10.1093/cid/ciaa556 32392337 PMC7239259

[5] Riphagen S Gomez X Gonzalez-Martinez C Wilkinson N Theocharis P. Hyperinflammatory shock in children during COVID-19 pandemic. Lancet. (2020) 395:1607–8. 10.1016/S0140-6736(20)31094-1 32386565 PMC7204765

[6] Royal College of Paediatrics and Child Health. Guidance: Paediatric Multisystem Inflammatory Syndrome Temporally Associated With COVID-19. (2020). Available online at: https://www.rcpch.ac.uk/sites/default/files/2020-05/COVID-19-Paediatric-multisystem-%20inflamma tory%20syndrome-20200501.pdf (accessed August 28, 2020).

[7] Verdoni L Mazza A Gervasoni A Martelli L Ruggieri M Ciuffreda M. An outbreak of severe Kawasaki-like disease at the Italian epicentre of the SARS-CoV-2 epidemic: an observational cohort study. Lancet. (2020) 395:1771–8. 10.1016/S0140-6736(20)31103-X 32410760 PMC7220177

[8] Belhadjer Z Méot M Bajolle F Khraiche D Legendre A Abakka S. Acute heart failure in multisystem inflammatory syndrome in children (MIS-C) in the context of global SARS- CoV-2 pandemic. Circulation. (2020) 142:429–36. 10.1161/CIRCULATIONAHA.120.048360 32418446

[9] Centers for Disease Control and Prevention. Multisystem Inflammatory Syndrome in Children (MIS-C) Associated With Coronavirus Disease 2019 (COVID-19). (2020). Available online at: https://emergency.cdc.gov/han/2020/han00432.asp (accessed January 25, 2021).

[10] World Health Organization. Multisystem inflammatory syndrome in children and adolescents with COVID-19: Scientific Brief. (2020). Available online at: https://www.who.int/publications/i/item/multisystem-inflammatory-syndrome-in-children-and-adolescents-with-covid-19 (accessed January 25, 2021).

[11] Levin M. Childhood multisystem inflammatory syndrome-a new challenge in the pandemic. N Engl J Med. (2020) 383:393–5. 10.1056/NEJMe2023158 32598829 PMC7346677

[12] Centers for Disease Control and Prevention. Health Department-Reported Cases of Multisystem Inflammatory Syndrome in Children (MIS-C) in the United States. (2020). Available online at: https://www.cdc.gov/mis-c/cases/index.html (accessed January 26, 2021).

[13] Kest H Kaushik A DeBruin W Colletti M Goldberg D. Multisystem Inflammatory Sindrome in Children (MIS-C) Associated with 2019 Novel Coronavirus (SARS-CoV-2) Infection. Case Rep Pediatr. (2020) 2020:8875987. 10.1155/2020/8875987 32733733 PMC7383305

[14] Esposito S Principi N. Multisystem inflammatory syndrome in children related to SARS-CoV-2. Paediatr Drugs. (2021) 22:1–11. 10.1007/s40272-020-00435-x PMC7819738 33479801

[15] Yasuhara J Watanabe K Takagi H Sumitomo N Kuno T. COVID-19 and multisystem inflammatory syndrome in children: A systematic review and meta-analysis. Pediatr Pulmonol. (2021) 11:25245. 10.1002/ppul.25245 33428826 PMC8013394

[16] McMurray JC May JW Cunningham MW Jones OY. Multisystem Inflammatory Syndrome in Children (MIS-C), a post-viral myocarditis and systemic vasculitis-a critical review of its pathogenesis and treatment. Front Pediatr. (2020) 8:626182. 10.3389/fped.2020.626182 33425823 PMC7793714

[17] Buonsenso D Riitano F Valentini P. Pediatric inflammatory multisystem syndrome temporally related with SARS-CoV-2: immunological similarities with acute rheumatic fever and toxic shock syndrome. Front Pediatr. (2020) 8:574. 10.3389/fped.2020.00574 33042918 PMC7516715

[18] Kaushik A Gupta S Sood M Sharma S Verma S. A systematic review of multisystem inflammatory syndrome in children associated with SARS-CoV-2 infection. Pediatr Infect Dis J. (2020) 39:e340–6. 10.1097/INF.0000000000002888 32925547

[19] Nelson C Ishimine P Hayden SR Correia M Wardi G. Multisystem inflammatory syndrome in children (mis-c) in an adolescent that developed coronary aneurysms: a case report and review of the literature. J Emerg Med. (2020) 59:699–704. 10.1016/j.jemermed.2020.09.008 33011038 PMC7527793

[20] Blondiaux E Parisot P Redheuil A Tzaroukian L Levy Y Sileo C. Cardiac MRI in children with multisystem inflammatory syndrome associated with COVID-19. Radiology. (2020) 297:E283–8. 10.1148/radiol.2020202288 32515676 PMC7294821

[21] Tandon R. Rheumatic fever pathogenesis: approach in research needs change. Ann Pediatr Cardiol. (2012) 5:169–78. 10.4103/0974-2069.99621 23129908 PMC3487207

[22] Barra SN Providencia R Paiva L Nascimento J Marques AL. A review on advanced atrioventricular block in young or middle-aged adults. Pacing Clin Electrophysiol. (2013) 35:1395–405. 10.1111/j.1540-8159.2012.03489.x 22897386

[23] Peigh G Leya MV Baman JR Cantey EP Knight BP Flaherty JD. Novel coronavirus 19 (COVID-19) associated sinus node dysfunction: a case series. Eur Heart J Case Rep. (2020) 4:1–6. 10.1093/ehjcr/ytaa132 33089039 PMC7239209

[24] Wang D Hu B Hu C Zhu F Liu X Zhang J. Clinical characteristics of 138 hospitalized patients with 2019 novel coronavirus-infected pneumonia in Wuhan, China. JAMA. (2020) 323:1061–9. 10.1001/jama.2020.1585 32031570 PMC7042881

[25] Al-Assaf O Mirza M Musa A. Atypical presentation of COVID-19 as subclinical myocarditis with persistent high-degreee atrioventricular block treated with pacemaker implant. Heart Rhythm Case Rep. (2020) 6:884–7. 10.1016/j.hrcr.2020.09.003 32953452 PMC7490251

[26] Forrester JD Mead P. Third-degree heart block associated with Lyme carditis: review of published cases. Clin Infect Dis. (2014) 59:996–1000. 10.1093/cid/ciu411 24879781

[27] Zheng YY Ma YT Zhang JY Xie X. COVID-19 and the cardiovascular system. Nat Rev Cardiol. (2020) 17:259–60. 10.1038/s41569-020-0360-5 32139904 PMC7095524

[28] El-Assaad I Hood-Pishchany MI Kheir J Mistry K Dixit A Halyabar O. Complete heart block, severe ventricular dysfunction, and myocardial inflammation in a child with COVID-19 infection. JACC Case Rep. (2020) 2:1351–5. 10.1016/j.jaccas.2020.05.023 32838330 PMC7250756

[29] Dionne A Mah DY Son MBF Lee PY Henderson L Baler AL. Atrioventricular block in children with multisystem inflammatory syndrome. Pediatrics. (2020) 146:e2020009704. 10.1542/peds.2020-009704 32855347

[30] Henderson LA Canna SW Friedman KG Gorelik M Lapidus SK Bassiri H. American college of rheumatology clinical guidance for pediatric patients with multisystem inflammatory syndrome in children (MIS-C) associated with SARS-CoV-2 and hyperinflammation in COVID-19. Version 2. Arthritis Rheumatol. (2020) 73:e13. 10.1002/art.41616 PMC8559788 33277976

[31] Cattalini M Taddio A Bracaglia C Cimaz R Paolera SD Filocamo G. Rheumatology study group of the italian society of pediatrics. childhood multisystem inflammatory syndrome associated with COVID-19 (MIS-C): a diagnostic and treatment guidance from the rheumatology study group of the Italian society of pediatrics. Ital J Pediatr. (2021) 47:24. 10.1186/s13052-021-00980-2 33557873 PMC7868856

[32] Toubiana J Poirault C Corsia A Bajolle F Fourgeaud J Angoulvant F. Kawasaki-like multisystem inflammatory syndrome in children during the covid-19 pandemic in Paris, France: prospective observational study. BMJ. (2020) 369:m2094. 10.1136/bmj.m2094 32493739 PMC7500538

[33] Burns JC Franco A. The immunomodulatory effects of intravenous immunoglobulin therapy in Kawasaki disease. Expert Rev Clin Immunol. (2015) 11:819–25. 10.1586/1744666X.2015.1044980 26099344 PMC4985263

[34] Cavalli G De Luca G Campochiaro C Della-Torre E Ripa M Canetti D. Interleukin-1 blockade with high-dose anakinra in patients with COVID-19, acute respiratory distress syndrome, and hyperinflammation: a retrospective cohort study. Lancet Rheumatol. (2020) 2:e325–31. 10.1016/S2665-9913(20)30127-2 32501454 PMC7252085

