# Supplementary figures and images for: Case Report: Mitral Valve Involvement and First-Degree Atrial-Ventricular Block in Two Patients With Multisystem Inflammatory Syndrome in Children
Source: Front Pediatr. 2021 Aug 6;9:676934. doi: 10.3389/fped.2021.676934 (PMC8377535; doi:10.3389/fped.2021.676934)

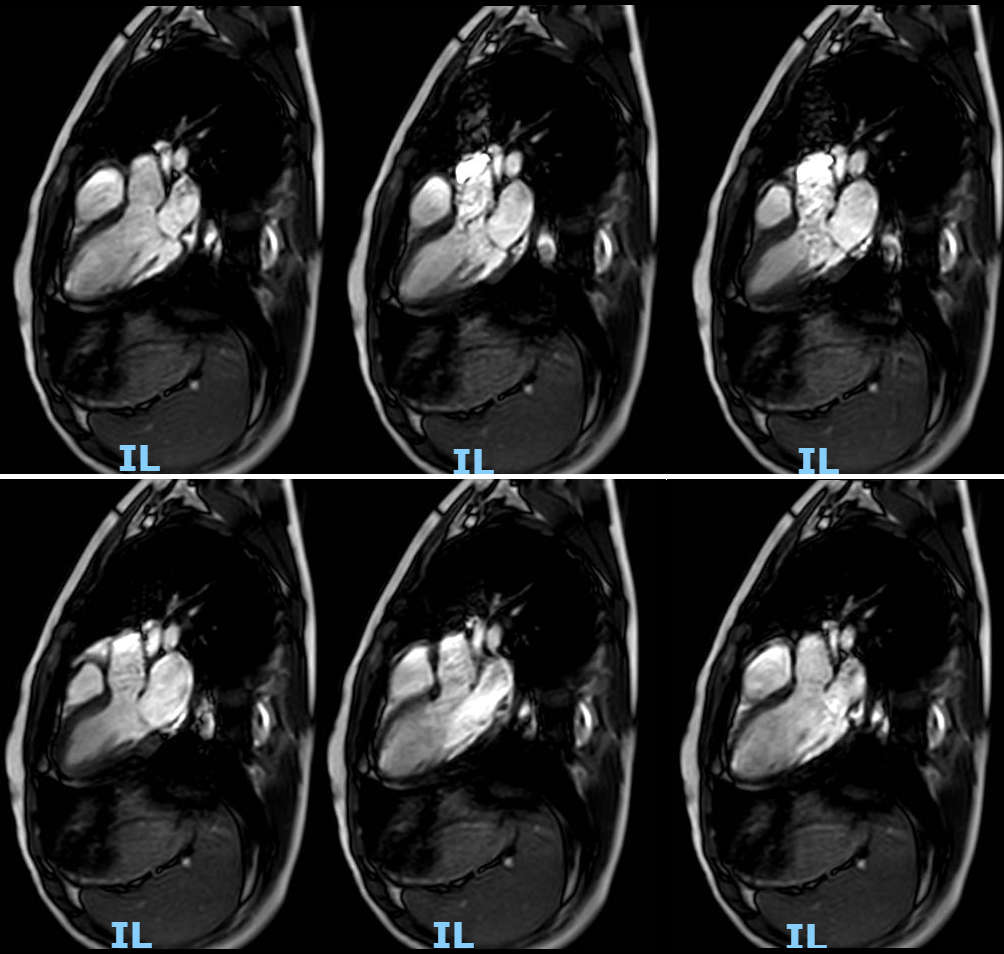

Supplement: Supplementary Figure 1 — The cardiac magnetic resonance imaging performed about 40 days after the onset found no morpho-functional abnormalities with non-dilated ventricles, a normal systolic function and a mild pericardial effusion with a maximum thickness of 7 mm. [file Image_1.PNG]
